# Vibrio cholerae outbreak in Italy.

**DOI:** 10.3201/eid0502.990221

**Published:** 1999

**Authors:** L Cavalieri d'Oro, E Merlo, E Ariano, M G Silvestri, A Ceraminiello, E Negri, C La Vecchia


### An Outbreak of Gastroenteritis in Japan due to *Escherichia coli* O166

**To the Editor:** Enteroaggregative *Escherichia coli* (EAggEC) heat-stable enterotoxin 1 (EAST1) was originally found as an enterotoxin of EaggEC (1). Recently, Yamamoto et al. (2) reported that the *EAST1* gene, or its variants, were present not only in EAggEC but in other diarrheagenic *E. coli,* including some enteropathogenic *E. coli* (EPEC) and enterotoxigenic *E. coli* (ETEC). Hedberg et al. (3) found that an outbreak of gastrointestinal illness in 1991 had been caused by EAST1-producing *E. coli* that possessed the EPEC gene locus for enterocyte effacement. We propose that *E. coli* producing EAST1 but possessing no other identifiable pathogenic properties may compose either a new group of diarrhea-associated *E. coli* or a new subgroup of ETEC.

In an outbreak of gastroenteritis on July 23, 1996, in Osaka, Japan, 54 of 91 persons attending a meeting held in an office building on July 22, 1996, became ill. The patients did not eat any common foods except the lunch served at the office. Symptoms were diarrhea in 52 (96%); abdominal pain in 32 (59%); nausea in 8 (15%); fever in 8 (15%); and vomiting in 5 (10%). The mean incubation period was 17 hours.

Stool specimens of 33 patients were examined, and *E. coli* O166 with an unidentifiable H antigen were isolated from 29 specimens. Laboratory tests for other bacterial pathogens and viruses were negative. The isolates showed the same DNA banding pattern in pulsed-field gel electrophoresis after treatment with the restriction enzymes *Xba* I or *Not* I.

The *E. coli* O166 organisms did not adhere to HEp-2 cells in a localized, diffuse, or enteroaggregative manner and did not give mannose-resistant hemagglutination of human or bovine red blood cells. Although the organisms were further analyzed for expression of known ETEC colonization factors by a dot-blot assay using specific monoclonal antibodies, they did not express CFA/I, CS1, CS2, CS3, CS4, CS5, CS6, CS7, CS17, PCFO159, PCFO166, or CFA/III. In polymerase chain reaction (PCR) tests, the bacteria did not have coding genes for verocytotoxin of enterohemorrhagic *E. coli,* heat-labile, or heat-stable enterotoxin of ETEC, attachment and effacement (*eae*A) of EPEC, or invasion (*inv*E) of enteroinvasive *E. coli.* Consequently, they are not assigned to any of the recognized diarrheagenic groups of *E. coli*: EPEC, ETEC, enterohemorrhagic *E. coli,* enteroinvasive *E. coli*, EAggEC, and diffusely adhering *E. coli*. According to the PCR method of Yamamoto et al. (2), however, the organisms possessed the *EAST1* gene.

To our knowledge, this is the first report of an outbreak caused by EAST1-producing *E. coli* that did not have other well-characterized virulence genes. We believe that these strains should be assigned to a new subgroup of ETEC. Such strains would not be detected in most current surveys for diarrheagenic *E. coli*, as tests for EAST1 are rarely included. The role of EAST1 in pathogenicity has been controversial. We propose that diarrheal specimens be examined for EAST1-producing *E. coli* so that the distribution of these organisms worldwide can be determined.

**Yoshikazu Nishikawa,* Jun Ogasawara,* Anna Helander, † and Kosuke Haruki***
*Osaka City Institute of Public Health and Environmental Sciences, Osaka, Japan;
†Göteborg University, Sweden

### Acknowledgment
We thank Sylvia M. Scotland, who retired from the Laboratory of Enteric Pathogens, Central Public Health Laboratory, London, for her critical review of the manuscript.

### References
1. Savarino SJ, Fasano A, Robertson DC, Levine MM. Enteroaggregative *Escherichia coli* elaborate a heat-stable enterotoxin demonstrable in an in vitro rabbit intestinal model. J Clin Invest 1991;87:1450-5.
2. Yamamoto T, Wakisaka N, Sato F, Kato A. Comparison of the nucleotide sequence of enteroaggregative *Escherichia coli* heat-stable enterotoxin 1 genes among diarrhea-associated *Escherichia coli.* FEMS Microbiol Lett 1997;147:89-95.
3. Hedberg CW, Savarino SJ, Besser JM, Paulus CJ, Thelen VM, Myers LJ, et al. An outbreak of foodborne illness caused by *Escherichia coli* O39:NM, an agent not fitting into the existing scheme for classifying diarrheagenic *E. coli.* J Infect Dis 1997;176:1625-8.

### *Vibrio cholerae* Outbreak in Italy

**To the Editor:** On 16 June, the microbiology unit of the Hospital of Lodi communicated to the local public health unit that *Vibrio cholerae* had been isolated and identified by standard biochemical tests in stool samples of an outpatient whose clinical data were unknown. On the same day, we contacted and interviewed the patient to investigate risk factors for cholera. The patient reported abdominal pain starting on 6 June and then severe diarrhea (10 to 12 stools per day) until 13 June; on that day the patient went to his general practitioner, who gave him loperamide and suggested a coproculture. The patient never traveled to cholera-endemic areas; did not eat raw mussels, uncooked fish, or vegetables of uncertain origin or from cholera-infected areas; and did not swim in rivers or lakes. The patient reported that he ate a seafood salad in the canteen of his work place on 5 June and that three out of the four persons who ate the same kind of salad also had abdominal symptoms. Subsequently, the Istituto Superiore di Sanità (ISS) in Rome confirmed isolation of toxinogenic *V. cholerae* O1, biotype El Tor, serotype Ogawa, from stools from the index patient.

The canteen where the index patient had eaten the seafood salad was 1 of 17 supplied by a single cooking center that used a precooked, frozen, ready-to-eat product including shrimps, scallops, mussels, hen clams, cuttlefishes, and squid. Each product was cooked and frozen in the country of origin and mixed in Italy by an importer who packaged the seafood salad. Tracking the products around the world was difficult, but we learned that at least some had come from Far East countries where cholera is endemic. Approximately 125 servings of the same food were distributed within our local public health area (Azienda Sanitaria Locale) and more than 400 in other areas.

We performed an epidemiologic case-control investigation beginning 18 June involving 454 persons (94 who had eaten the seafood salad and 360 controls who had eaten in the same canteen any food except seafood salad); 37 (39%) of the persons who had eaten the seafood salad had had at least one episode of diarrhea or other relevant gastrointestinal symptoms, as compared to one (0.3%) of those who had not eaten it. We did not find symptomatic patients. The corresponding odds ratio was 233 (95% confidence interval, 97 to 560). No symptomatic person had to be hospitalized because of symptoms or required intravenous treatment; three or more loose or watery stools during a 24-hour period were reported in 24 cases. We performed coprocultures (using TCBS medium) between 23 June and 3 July of all 94 persons who had eaten the seafood salad. One positive coproculture for *V. cholerae* O1 Ogawa (same strain) was identified on 25 June; the isolation was subsequently confirmed by the ISS. This second patient with a positive culture worked in a factory in a different town. She had severe diarrhea on 6, 7, and 8 June. Her family doctor gave her rifaximin but did not ask for a coproculture, but a specimen was obtained on 23 June. She did not report risk factors for cholera infection, except having eaten seafood salad on June 5 in the canteen of her work place. The delay between exposure to *V. cholerae* and the coprocultures was longer than 1 week (median delay 26 days, range 19 to 31), and it is therefore not surprising that others who had eaten the seafood salad did not have positive results. Both the culture-positive index case-patient and the woman were recultured three more times; negative results were obtained.

The identification of this cholera outbreak is a sentinel episode confirming (1,2) that, if not adequately monitored, food preparation and distribution can cause serious infectious diseases in industrialized countries.

**Luca Cavalieri d'Oro,*† Elisabetta Merlo,† Eugenio Ariano,† Maria Grazia Silvestri,† Antonio Ceraminiello,† Eva Negri,* and Carlo La Vecchia*‡**

*Istituto di Ricerche Farmacologiche "Mario Negri," Milan, Italy; †Azienda Sanitaria Locale della Provincia di Lodi, Lodi, Italy; and ‡Università degli Studi di Milano, Milan, Italy

### References

1. Centers for Disease Control and Prevention. Cholera associated with food transported from El Salvador—Indiana, 1994. MMWR Morb Mortal Wkly Rep 1995;44:385-6.
2. Centers for Disease Control. Cholera associated with imported frozen coconut milk—Maryland, 1991. MMWR Morb Mortal Wkly Rep 1991;40:844-5.

### Shiga Toxin–Producing *Escherichia coli* O157:H7 in Japan

**To the Editor:** Shiga toxin-producing *Escherichia coli* (STEC) O157:H7 infection, which can cause hemolytic uremic syndrome and death, is a global public health concern. Patients younger than 5 years of age are at high risk for hemolytic uremic syndrome (1) and shed the organism longer than adults (2). The public health

